# Urinary incontinence and its association with functional physical and cognitive health among female nursing home residents in Switzerland

**DOI:** 10.1186/s12877-017-0414-7

**Published:** 2017-01-13

**Authors:** Lea F. Schumpf, Nathan Theill, David A. Scheiner, Daniel Fink, Florian Riese, Cornelia Betschart

**Affiliations:** 1 Department of Gynecology, University Hospital Zurich, Frauenklinikstrasse 10, 8091 Zurich, Switzerland; 2 University Research Priority Program “Dynamics of Healthy Aging”, University of Zurich, Zurich, Switzerland; 3 Psychiatric University Hospital Zurich, Division of Psychiatry Research and Division of Psychogeriatric Medicine, Zurich, Switzerland

**Keywords:** Urinary incontinence, Nursing home, Activities of daily living (ADL), Cognitive performance scale (CPS), Comorbidities

## Abstract

**Background:**

Specific knowledge of urinary incontinence (UI) and its interrelation with physical and cognitive health is essential to working towards prevention of UI and to improving quality of treatment and care. The purpose of this study was to determine the association between UI and the activities of daily living (ADL) hierarchy scale, the cognitive performance scale (CPS) and comorbid conditions.

**Methods:**

The cross-sectional retrospective analysis of 357 nursing homes in Switzerland was based on data of the Minimum Data Set of the Resident Assessment Instrument 2.0 (RAI-MDS). The analysis examined the effect of ADL hierarchy scale, CPS, joint motion and comorbidities on UI. Wome*n* ≥65 years were included (*n* = 44’811; January 2005 to September 2014) at the time of admission to a nursing home. Statistical analysis was done by means of descriptive statistics and logistic regression analysis.

**Results:**

The prevalence of UI was 54.7%, the mean ADL hierarchy scale (± SD) 2.42 ± 3.26 (range = 0–6), the mean CPS 1.95 ± 1.67 (range = 0–6). There was a gradual increase in the odds ratio (OR) for UI depending on the ADL hierarchy scale, from the hierarchy scales of “supervised” to “total dependence” of 1.43 – 30.25. For CPS, the OR for UI from “borderline intact” to “very severe impairment” was 1.35 – 5.99. Considering the interaction between ADL and CPS, all ADL hierarchies remained significantly associated with UI, however for CPS this was the case only in the lower hierarchies. Of the 11 examined comorbid conditions, only diabetes mellitus (OR 1.19), dementia (OR 1.01) and arthrosis/arthritis (OR 1.53) were significantly associated with UI.

**Conclusion:**

The study indicated that impairment in ADL performance is strongly associated with UI, more than CPS performance and comorbidities. Physical more than cognitive training in order to improve or at least stabilize ADL performance could be a way to prevent or reduce the process of developing UI.

## Background

Urinary incontinence (UI) has a profound impact on an affected woman’s life. It is associated with a significantly reduced health-related quality of life [1] and depression [2, 3]. Old people with UI are more dependent on professional services [4]. UI impairs the person’s independence and can trigger nursing home admission, even though data whether UI is a predicting factor for nursing home admission were inconsistent in a systematic review [5]. Furthermore, UI is a predictor of death and mortality rate increases in parallel with UI severity, as shown in a group of community-dwelling participants receiving home care services [4]. Beside this individual burden, UI is associated with higher healthcare resource utilization [1]. The annual direct costs of UI in the United States were estimated at US$ 19.5 billion in 2000, with US$ 5.3 billion for institutionalized old people [6]. With an aging population in Western countries [7], the prevalence of UI and associated problems is increasing rapidly [8]. Studies which define UI as *any involuntary leakage of urine* showed a prevalence between 72 and 78% in women living in nursing homes [9].

UI is often multifactorial in etiology; next to physiological age-related changes in lower urinary tract function, risk factors outside the lower urinary tract, such as co-existing disabilities and comorbidities, are causing or contributing to UI [10–12]. Specific knowledge and awareness of UI and its interrelation with physical and cognitive health is essential to working towards prevention of UI and to improving quality of treatment and care.

Hence, the purpose of this study was to identify whether dependency on activities of daily living, cognitive performance and comorbid conditions are associated and interrelated with UI in women at the time of nursing home admission.

## Methods

### Study design and data source

**Fig. 1:**
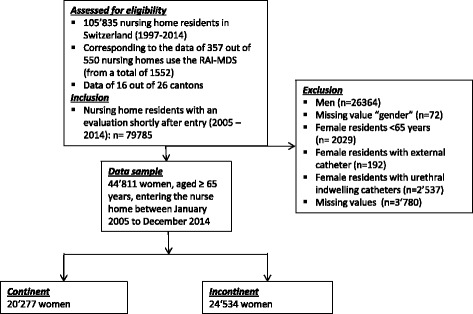
Derivation of Study sample

This is a cross-sectional secondary analysis of the healthcare data from the Minimum Data Set (MDS) of the Swiss Version of the Resident Assessment Instrument (RAI) 2.0 [13, 14]. The study sample was drawn of a dataset of 105’835 nursing home residents in Switzerland with at least one MDS assessment as shown in the flow chart of Fig. 1. The dataset was provided by the local distribution and administration company of the RAI system, Q-Sys AG, St. Gallen, which also obtained the authorization from the participating nursing homes. All personal information was removed before data export, so no approval from the local ethics committee was required (cantonal ethics committee Zurich declaration of no objection 103–2015, KEK-ZH-Nr. 2012–0102). A group comparison of women without and with indwelling catheter was done in a subgroup analysis.

### Instruments

The RAI-MDS is a widely used and standardized instrument for the assessment of the health status and care needs of old people living in a nursing home [15]. In Switzerland, there are 1552 nursing homes, 550 of which are using the RAI-System [13]. The assessments are carried out by nursing staff in cooperation with other healthcare professions shortly after nursing home admission and at periodic intervals of 6 months or whenever a significant status change occurs [13]. A large proportion of the items in the RAI achieves an adequate to excellent level of reliability, with no substantial differences across countries [16].

### Measures

#### Urinary incontinence (UI)

The RAI-MDS item on bladder incontinence was used to identify female nursing home residents with UI at their age of admission. A five-point coding scale is used to describe continence patterns depending on frequency of leakage (level 0 = no incontinence, level 4 = incontinent most of the time). For our analysis, the definition of UI recommended by the International Continence Society (ICS) was utilized, which considers any involuntary leakage of urine [17]. Therefore all non-zero values (level 1–4) were coded as UI.

In the RAI-MDS 2.0, bladder control is recorded with appliances (for example bladder catheter), thus nursing home residents presenting with a bladder catheter or ostomy were excluded from analysis (see Fig. 1), because the reasons for continuous bladder draining may vary heterogeneously from bladder atonia to complete incontinence.

#### Activities of daily living (ADL)

The ADL Hierarchy Scale derived from the RAI-MDS was used to assess functional physical health. The ADL Hierarchy Scale has been shown to be a valid measure for ADL in nursing home residents. This scale is based on four items (personal hygiene, use of toilet, locomotion and eating) and early-loss ADL (for example personal hygiene) are assigned lower scores than late-loss ADL (for example eating). It has seven levels ranging from total independence (level 0) to total dependence (level 6) [18].

#### Cognitive performance scale (CPS)

Cognitive function was measured using the Cognitive performance scale (CPS). The CPS combines five selected items of the RAI-MDS within a hierarchical seven-category rating scale, ranging from no cognitive impairment (level 0) to very severe impairment (level 6). The five MDS items used to construct the CPS include two cognitive measures (short-term memory, cognitive skills for daily decision making), one communication measure (making self-understood), one ADL measure (eating), and comatose status [19]. The CPS corresponds closely to Mini-Mental State Examination scores [19, 20].

#### Limitation in range of joint motion and loss of voluntary movement

In the RAI-MDS, the presence of limitation in range of joint motion and loss of voluntary movement is recorded by each body part (0 = no limitation/no loss of voluntary movement, 1 = limitation on one side of the body/partial loss of voluntary movement, 2 = limitation on both sides of the body/full loss of voluntary movement). In this work, we focused on arm, hand, leg and foot in order to detect whether a difference in the prevalence of UI exists relative to the functional impairment of upper or lower extremities.

#### Comorbidities

In the RAI-MDS, diseases or infections are coded that have a relationship to the resident’s current ADL status, cognitive status, mood or behaviour status, medical treatments, nursing monitoring or risk of death. The noted disease conditions require a physician-documented diagnosis in the clinical record. In this study, we focused on the following comorbidities: diabetes mellitus, arteriosclerosis, heart disease, cardiac dysrhythmias, congestive heart failure, hypertension, arthrose/arthritis, osteoporosis, dementia, cerebrovascular accident, anxiety disorder, depression, asthma, emphysema/COPD, chronic urinary tract infection and renal failure.

### Statistical analyses

Descriptive statistics were calculated for all measures. Logistic regression was performed to assess the impact of residents’ age at admission, ADL hierarchy, CPS, limitation in range of motion, and comorbidities on UI at the first assessment after admission to a nursing home. In a first step, a model with all selected variables included was calculated. Thereafter, the effect of every single predictor variable on the model was tested to exclude all predictor variables that do not contribute to the model and to get a more parsimonious model. As a result, the predictors of the final model were reduced to age at admission, ADL hierarchy, CPS, diabetes, arthrosis and dementia. However, as the Hosmer-Lemeshow test indicated poor model fit due to potential misspecification of the model, interaction between ADL hierarchy and CPS was additionally considered. All statistical analyses were calculated with SPSS 21 (SPSS Inc., Chicago, Il.) for Macintosh, using a significance level of α = .001.

## Results

### Subjects’ characteristics

Among the 44’811 eligible women (Fig. 1), 24’534 had some level of UI upon admission with a prevalence of 54.7%. The sample mean age (± SD) was 84.6 ± 6.9 years. More than 80% of all examined women were to some extent dependent regarding ADL and 50.9% needed extensive care or complete support; mean ADL hierarchy scale (± SD) was 2.42 ± 3.26 (range = 0–6). Fifty-five percent of the included sample had mild to very severe cognitive impairments, with a mean CPS score (± SD) of 1.95 ± 1.67 (range = 0–6). The mean number of comorbid conditions (± SD) was 2.94 ± 1.75 (range = 0–11).

**Fig. 2:**
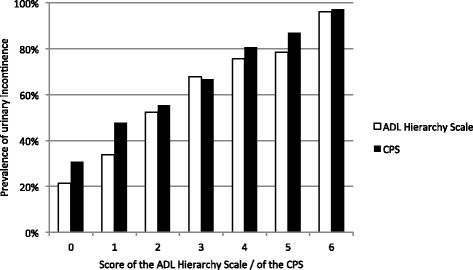
Association between prevalence of urinary incontinence and the ADL hierarchy scale and the CPS. Higher numbers in ADL and CPS indicate higher levels of impairment (zero = unimpaired)

Characteristics of continent versus incontinent female nursing home residents at the time of admission are shown in Table 1. The prevalence of UI increased with age, higher ADL dependency and more severe cognitive impairment (Table 1, Fig. 2), as well as with higher limitation in range of joint motion / more loss of voluntary movement and the number of comorbid conditions (Table 1).

**Table 1:** Characteristics of continent versus incontinent females upon admission to a nursing home

	All	No UI	UI
	*n* = 44’811	*n* = 20’277	*n* =24’534
All	100%	45.3%	54.7%
Age			
65– 74	8.7%	51.8%	48.2%
75 – 84	37.2%	48.2%	51.8%
85 – 94	48.3%	43.1%	56.9%
>94	5.8%	34.4%	65.6%
ADL Hierarchy			
0 (Independent)	19.0%	78.7%	21.3%
1 (Supervision)	10.9%	66.2%	33.8%
2 (Limited)	19.3%	47.6%	52.4%
3 (Extensiv 1)	25.5%	32.5%	67.5%
4 (Extensiv 2)	12.4%	25.1%	74.9%
5 (Dependent)	11.4%	21.9%	78.1%
6 (Total dependence)	1.6%	3.9%	96.1%
Cognitive performance scale			
0 (Intact)	26.9%	69.2%	30.8%
1 (Borderline Intact)	18.1%	52.1%	47.9%
2 (Mild Impairment)	15.7%	44.7%	55.3%
3 (Moderate Impairment)	24.2%	33.4%	66.6%
4 (Mod. Severe Impairment)	3.8%	19.6%	80.4%
5 (Severe Impairment)	9.7%	13.5%	86.5%
6 (Very Severe Impairment)	1.6%	2.9%	97.1%
Number of comorbidites			
0	5.8%	56.9%	43.1%
1	14.9%	49.0%	51.0%
2	23.0%	46.3%	53.7%
3	22.6%	44.7%	55.3%
4	15.9%	41.8%	58.2%
5	9.4%	41.2%	58.8%
6	5.0%	39.9%	60.1%
7	2.1%	41.9%	58.1%
8	0.8%	36.1%	63.9%
9	0.3%	41.6%	58.4%
10	0.1%	33.3%	66.7%
11 (*n* = 13)	0.0%	23.1%	76.9%
Limitation in rang of join motion			
Arm			
0 (no limitation)	71.2%	49.3%	50.7%
1 (limitation on one side)	15,4%	40.9%	59.1%
2 (limitation on both side)	13.3%	28.6%	71.4%
Hand			
0 (no limitation)	82.5%	47.8%	52.2%
1 (limitation on one side)	9.3%	37.3%	62.7%
2 (limitation on both side)	8.2%	28.1%	71.9%
Leg			
0 (no limitation)	64.8%	51.1%	48.9%
1 (limitation on one side)	16.4%	42.6%	57.4%
2 (limitation on both side)	18.8%	27.3%	72.7%
Foot			
0 (no limitation)	77.9%	49.5%	50.5%
1 (limitation on one side)	9.0%	38.5%	61.5%
2 (limitation on both side)	13.1%	24.7%	75.3%
Loss of voluntary movement			
Arm			
0 (no loss)	70.9%	49.7%	50.3%
1 (partial loss)	26.4%	35.2%	64.8%
2 (full loss)	2.7%	27.1%	72.9%
Hand			
0 (no loss)	81.8%	48.20%	51.8%
1 (partial loss)	16.0%	33.20%	66.8%
2 (full loss)	2.2%	23.10%	76.9%
Leg			
0 (no loss)	63.9%	51.8%	48.2%
1 (partial loss)	33.1%	34.6%	65.4%
2 (full loss)	3.1%	22.8%	77.2%
Foot			
0 (no loss)	77.1%	49.8%	50.2%
1 (partial loss)	20.0%	31.1%	68.9%
2 (full loss)	3.0%	21.6%	78.4%

About half of the women admitted to a nursing home with no limitation in range of joint motion and no loss of voluntary movement by arm, hand, leg or foot were affected by UI. The prevalence of UI increased similarly with more functional impairment of upper and of lower extremities.

### Odds for UI

The logistic regression model with the six selected predictor variables explained 30.1% (Nagelkerke R squared) of the variance in UI, and 71.3% of all cases were correctly classified.

The variables of “joint motion of upper and lower extremities” were excluded in the process of model fitting. In a first model, where all selected variables were included, the analysis of the limitation in range of motion (reference: limitation on both sides of the body) showed a significant reduction in the OR in “limitation on one side of the body” related to leg (OR 0.750, CI 0.69 – 0.82) or foot (OR 0.842, CI 0.73 –0.93). Apart from that, no more significant OR by upper as well as by lower extremities was found.

A further model, including the relevant variables only, revealed an age-related difference (division of the examined female residents into age groups 65–74, 75–84, 85–94, ≥94 years) in UI. The odds to be incontinent were 20% higher for a female resident with an age between 75 and 84 years and 75% higher for an over 94 years old female resident compared with somebody with an age between 65 and 74 years. Of all comorbid conditions only diabetes mellitus, dementia and arthrosis/arthritis were relevant (see Table 2). Diabetes mellitus was found to be most important with an OR of 1.189 (CI 1.12 – 1.26). The variables “cerebrovascular accident” and “chronic urinary tract infection” were initially significant, but they were excluded after the model fitting process.

**Table 2:** Logistic regression analysis for urinary incontinence with ADL hierarchy, CPS, age, and comorbidities as predictors (model 1), and additional interaction between ADL hierarchy and CPS (model 2, interaction terms not shown in table). In both models, age 65–74, ADL hierarchy of 0, CPS score of 0, and having none of the tested diseased served as reference categories

	Model 1			Model 2		
	Odds ratio	95% CI	p-value	Odds ratio	95% CI	p-value
ADL Hierarchy						
0 (Independent)	Reference			Reference		
1 (Supervision)	1.238	1.14–1.35	<.001	1.427	1.22–1.69	<.001
2 (Limited)	2.723	2.54–2.92	<.001	2.232	1.99–2.50	<.001
3 (Extensiv 1)	4.701	4.39–5.04	<.001	3.293	2.95–3.68	<.001
4 (Extensiv 2)	6.209	5.71–6.76	<.001	3.831	3.29–4.46	<.001
5 (Dependent)	7.364	6.74–8.05	<.001	4.353	3.72–5.09	<.001
6 (Total dependence)	16.598	10.3–26.9	<.001	30.25	3.70–248	0.001
Cognitive performance scale						
0 (Intact)	Reference			Reference		
1 (Borderline Intact)	1.619	1.52–1.72	<.001	1.349	1.18–1.54	<.001
2 (Mild Impairment)	2.217	2.07–2.37	<.001	1.406	1.21–1.63	<.001
3 (Moderate Impairment)	2.975	2.78–3.19	<.001	1.287	1.05–1.59	0.170
4 (Mod. Severe Impairment)	4.990	4.36–5.72	<.001	1.338	0.48–3.70	0.575
5 (Severe Impairment)	6.902	6.20–7.68	<.001	1.599	0.74–3.46	0.233
6 (Very Severe Impairment)	12.593	7.31–21.7	<.001	5.988	0.68–52.5	0.106
Age						
65– 74	Reference			Reference		
75 – 84	1.201	1.11–1.30	<0.001	1.189	1.10–1.29	<0.001
85 – 94	1.452	1.34–1.57	<0.001	1.409	1.30–1.53	<0.001
> 94	1.754	1.56–1.97	<0.001	1.677	1.49–1.89	<0.001
Comorbidities (Reference not diseased)						
Diabetes Mellitus	1.189	1.12–1.26	<0.001	1.181	1.12–1.25	<0.001
Arthrose/Arthritis	1.530	1.10–1.21	<0.001	1.155	1.10–1.21	<0.001
Dementia	1.090	1.04–1.16	<0.001	1.107	1.05–1.17	<0.001

The range of OR for UI from the old person being “supervised” to “total dependence” in ADL performance was 1.238 – 16.598. For the CPS, the range of OR from “borderline intact” to “very severe impairment” was 1.619 – 12.593 (Table 2, model 1).

Additionally, the interaction between the variables was tested. There was significant interaction between the ADL hierarchy scale and the CPS. Therefore, a further model was calculated considering the interaction between these two scales (Table 2, model 2). This model explained 31.1% (Nagelkerke R squared) of the variance in UI, with correct classification of 71.6% of cases. The significant effect of CPS on UI seen in model 1 has thus to be relativized. A borderline intact (OR 1.349, CI 1.18 – 1.54) or mildly impaired cognitive performance (OR 1.406, CI 1.21 – 1.63) was significant, but a more severe level of cognitive impairment was no longer significantly associated with UI. With increasing cognitive impairment, the initial effect of cognitive impairment on UI disappears and is explained by its interaction with ADL dependency. Although increase in total variance was comparably low for the model with interaction (considering the number of additional parameters estimated), the significant Hosmer-Lemeshow test in the model without interaction indicated a low model fit and those results should be interpreted with caution. However, it has to be noticed that the test is sensitive to large sample sizes.

When adjusted by the interaction between the ADL hierarchy scale und the CPS, higher levels of ADL dependence were still related to a higher OR. The odds to be UI for a female resident who was limited or dependent in ADL was more than 2 times respectively more than 4 times higher than for an ADL independent female resident. As there were fewer cases in the highest groups (ADL hierarchy of total dependence and CPS = 6 or “very severe impairment”) with some non-existing combinations (e.g. a CPS score of 6 is always associated with dependency or total dependency), leading to large standard errors for those estimates especially in the model with interaction (see Table 2), the two highest groups were aggregated (ADL hierarchy = 5 and 6, and CPS = 5 and 6) in a further model, which provided almost identical estimates and model fits, both for the model with and without interaction and thus is not reported in this paper. However, there is of course no longer a unique effect for total dependence or severe impairment in this model, as those cases are placed in the dependent and severe impairment group, respectively. Women with an indwelling urinary catheter (*n* = 2538), excluded from the main analysis, were younger (mean age of 83.2 ± 7.3 years), more comorbid (comorbidities of 3.29 ± 1.92) and more impaired according to the ADL (mean 4.35 ± 1.10) and CPS scale (2.59 ± 1.92) compared to the main collective (student’s *t-*test, *p* < 0.001).

## Discussion

### Activities of daily living and UI

This study demonstrates a strong and gradual relation between ADL performance and UI among women at the time of nursing home admission in a large sample size. The ADL performance outweighed other factors like the CPS and comorbidities. An association between UI and ADL performance [2, 21] was part of other non-gender-specific analyses, however not tested in additional interaction models as presented. One cross-sectional study [21] showed that being incontinent led to an average MDS-ADL long form (ranging from 0 = (maximal independence) to 28 (maximal dependence)) value 3.81 points higher than in a continent person and in a survival analysis continence was a protective factor against ADL deterioration [22]. Another cross-sectional study established that more severe levels of UI were associated with more ADL impairment and that urinary-incontinent nursing home residents had impaired CPS scores, were hospitalized more frequently and had more urinary tract infections, pressure ulcers and depression than their continent counterparts [2]. Our study extends these findings by presenting relevant differences in the prevalence of UI depending on the level of ADL performance.

An association between UI and mobility restriction among older people was previously described [23, 24]. In a cohort of community-dwelling women it was demonstrated that participants with daily UI had 3.31 times increased odds and women with some (weekly, monthly, or yearly) UI had 1.61 times increased odds of functional difficulty or dependence compared with older women without UI [25]. It was also shown that nursing home residents with UI more often required a wheelchair [2] or walking aids [23].

These findings suggest that either UI can lead to a decrease in physical activity, which has a negative impact on the ADL, or that ADL deterioration and compromised mobility affect UI due to the inability to reach the restroom timely. We wanted to further clarify the direction of this association assuming that women with lower extremity impairment have more UI, and investigated the difference in prevalence of UI between upper and lower extremity impairment. It could be demonstrated that preservation of a good strength of lower extremities was associated with a better performance in ADL [26]. However, in our analysis, the effect of higher ADL on UI was not strongly associated with a difference in joint motion or loss of voluntary movement between the upper or lower extremities. Only a moderate association to being less incontinent was found for cases where the limitation in range of motion was related to only one leg or one foot compared to being limited in both lower extremities (feet and legs).

In a cross-sectional analysis that examines residents at one point in time it is only possible to identify associations, not to assess causality or directionality between ADL and UI.

### Cognitive performance and UI

In our data, the effect of cognitive performance on UI was mainly explained by the significant interaction between the ADL hierarchy scale and the CPS in the higher levels of dependence or impairment. Impairments in ADL had a much stronger influence on continence than cognitive impairments. However, after considering the interaction of these two scales, the calculated OR for UI was still significant in low levels of the CPS. The odds to be urinary incontinent for a woman admitted to a nursing home who had a borderline intact or a mildly impaired cognitive performance were 35% respectively 40% higher than for somebody without cognitive impairments.

A relation between UI and cognitive performance was previously found in non-gender-specific studies using the CPS [2] or other screening tests like the Pfeiffer test for cognitive status and the Barthel Index for functional capacity [23]. Furthermore, a significant difference in continence between residents with and without dementia was found by showing that the prevalence of UI was high even in early dementia (64%) and reaching 94% in severe dementia [27]. This data concurs with ours where 66.6% of women with moderate CPS impairment, respectively 97.1% with severe impairment, were incontinent. Those previous studies did not examine the interaction of ADL and CPS on UI, however. Similar to the relation between ADL performance and UI, the relation between UI and cognitive function might be bi-directional. So, limitations in ADL could prevent a person from using the toilet timely and could cause functional UI. Alternatively, UI could be caused by dysfunction of the frontal cortex in the context of neurodegenerative disease or stroke. The function of the micturition centre in the brain stem is under the control of the frontal lobe of the brain. A strong relation between UI and performance in cognitive tasks that reflect prefrontal cortex function has previously been demonstrated [28].

### Comorbidities and UI

An association between UI and medical comorbidities has been recognized in previous studies. However, variability exists; in our and other studies diabetes mellitus, dementia [2, 3, 27] and arthrosis/arthritis [3] were associated with a higher prevalence of UI. Other studies have shown that there was an association with heart failure [3, 29], depression [2, 3], stroke [23], hypertension [3] and asthma [29] — which was not found in our study. Co-morbidities can affect continence through multiple mechanisms. For example, diabetes mellitus may lead to polyuria and peripheral neurological damages that both affect the bladder function. Some drugs, for example cholinesterase inhibitors used for dementia, may cause or worsen UI [10]. Arthrosis/arthritis may contribute to the inability to access a toilet in a timely manner. Interestingly, the association between arthrosis/arthritis and incontinence on one side and the rather weak odds for UI and lower extremity impairment on the other side did not coincide strongly. In general, the effects of comorbidities on UI in our study were rather low, which also could be a result of a low or non-reporting bias.

However, the prevalence of UI increases with the number of comorbid conditions (not tested due to redundancy in the initial model with all comorbidities). In a cohort of community-dwelling persons a positive linear association between the number of comorbid conditions and the prevalence of UI was found [3]. The presence or absence of a particular individual comorbid disease was found to be less important than the cumulative effect of multiple diseases on physical health [12].

### Strengths and limitations

A strength of our study is its large sample size, which is much larger than in previous reported studies [2, 21, 23, 27, 28], and the assessment with the RAI-MDS that has also been shown to be a reliable instrument [16]. Unfortunately, the RAI-MDS does not contain information on type of UI (stress, urgency, or mixed UI) and drug treatment for UI wasn’t assessed either. The impact of some comorbidities such as heart failure or depression could have been underestimated due to underreporting in medical records. Due to the observational nature of the data, it is not possible to assess causality. However, cross-sectional analyses are useful because they can be a snapshot that leads to future research and hypothesis generation like the directionality of ADL and UI in a longitudinal study.

## Conclusion

UI is a highly prevalent health issue, affecting more than half of the women at the time of nursing home admission. UI is a multifactorial syndrome resulting from or exacerbated by several risk factors. Management of UI should focus on modification of risk factors among nursing home residents. Our study confirms for female nursing home residents the association between ADL [2, 21, 22], cognitive function [2, 23, 27], comorbid conditions [3, 12] and UI that has been shown in the literature. Our study adds the strength of correlation between UI and ADL performance that was stronger than with any other impairment or surrogate of impairment, like CPS, single comorbidities or disabilities of upper or lower extremities. UI as a geriatric symptom is strongly associated with the impairment of ADL that comprise physical and functional parameters. Furthermore, even mild ADL dependency and mild cognitive impairments were associated with a higher prevalence of UI, however there is insufficient evidence rule out effectiveness of interventions in people with cognitive impairment yet [30]. Physical more than cognitive training in order to improve or at least stabilize ADL performance could be a way to prevent or reduce the process of developing UI or vice versa [31, 32], potentially leading to improved quality of life and lower healthcare costs.
